# Annotations

**Published:** 1893-04-08

**Authors:** 


### Annotations.

**A Curious Survival at Tunbridge Wells.**

Last week the High Street of Tunbridge Wells was brilliant with innumerable gay equipages, prancing horses, and magnificently attired coachmen, and the quiet hospital of the town was for two or three hours almost as difficult of approach as if it had been a Royal Drawing Room. What was the attraction? An election by popular vote of a physician to fill the place of Dr. Johnson, retired. Now the election of a physician by popular vote seems about as wise a proceeding in these scientific times as the ordeal by poison which is employed in Central Africa in place of our own judge and jury. Yet at the Tunbridge Wells Hospital, which is an institution of considerable magnitude and importance, the whole of the subscribers are entitled to cast their votes for the medical officers, and the majority of votes carries the day. But, it may be asked, is there no previous selection and nomination of suitable candidates by a Medical Committee, or even by the General Committee of the hospital? None whatever! When a vacancy is declared, every medical man in Tunbridge Wells, who desires the post of physician to the hospital, prepares his circular and sends it out to the subscribers exactly as if he were a Parliamentary candidate asking for their votes, or a teadealer asking for their patronage in tea. On the voting day the supporters of the different candidates appear in force, and the most popular practitioner carries the day. Now this may be all very well from a popular point of view, but from a medical and scientific standpoint it is absurd. We venture to say that no medical man of really high scientific standing and just self-respect would submit his claims to such a tribunal. It may often happen that a man shall be compelled to submit himself and his claims to judges who have no knowledge or capacity for estimating his merits. When such compulsion is inevitable it must be endured with the best grace at command. But, undoubtedly, a high-minded physician would wait for ever for a hospital appointment rather than voluntarily choose to be judged by men and women who know no more of physic than they do of astronomy, and perhaps not so much. It is to us an astonishing circumstance that the hospital subscribers themselves do not insist upon the assistance of a medical committee, which shall at least see that positively unsuitable candidates are prevented from going to the poll. The whole thing is an anachronism, and worthy rather of an old-fashioned Sussex village than of a town like Tunbridge Wells, where persons of rank and wealth abound, and where intelligence also ought to abound.

**The Beginnings of Insanity.**

Authoritative pronouncements by trusted persons on this subject are of first-rate importance to all classes of brain-workers. In its early stages insanity is one of the most curable of all diseases, if the patient can be promptly placed under curative conditions. At the Medical Society's meeting on March 20th Dr. Savage read a paper on the "Symptoms of Mental Dissolution." Now "mental dissolution" is a euphuism for insanity, and Dr. Savage is deservedly one of our most trusted "alienists." Brain-workers should pay heed to what he said. This is what Dr. Savage said: "Loss of memory is the most common single symptom" which is approximately "pathognomonic" of the early stages of insanity. Let brain-workers pull themselves together then when they begin to be conscious of marked failure of memory. That is the time to consult a physician, a physician of clear judgment and absolute firmness of character; a physician who will rather lose his patient than yield to the giving of any advice except such as will at once result in the beginning of memory and health restoration for the patient. The next symptom, in the order of importance, is "loss of control." Loss of control announces itself in some persons by irritability of temper, in some by gusts of passion, in some by sexual excesses, and so on. Upon these follows a third symptom which is much less easy of detection both by the patient and his friends, the symptom of the loss of judgment. On the part of the patient the most marked indication of a loss of judgment is often a heightened self confidence, a more general and absolute determination to have his own way at any and every cost. The ordinary education even of educated persons does not fit them for understanding and properly valuing such facts as we have just set before readers. Many educated persons, especially writers, think they know better than the doctor. But the simple fact is that they do not; they really fail to comprehend the question. Physiology and physic are often as difficult as astronomy and the higher mathematics. A docile mind is one of the highest results of education and one of the most "pathognomonic" marks of sanity. The brain-worker who is already disposed to contemn the competent physician is on the borderland of that failure of judgment and loss of self-control which are among the early indications of insanity.

**Rags and Reason.**

"With respect to small-pox cases," says the *Times*, "Dr. Collingridge points out that many of the consignments of rags brought into the country are in an indescribably filthy condition, and are responsible for several outbreaks at different periods." If filthy rags have been responsible for outbreaks of small-pox, is it not at least conceivable that they may have been responsible for outbreaks of other diseases of the zymotic class? Undoubtedly it is; and the man who has even a small degree of passion for thoroughness begins to ask himself why, in the name of common sense, we should import cargoes of filthy and poisonous rags? Rags, perhaps, we must have; but why rags that poison the air and the workers with abominable and pestiferous smells? How are we to deprive the rags of their filthiness? The answer is, by boiling them in antiseptics. But where and by whom are they to be boiled in antiseptics? That is a detail which the traffickers in rags should themselves be compelled to deal with. They can be boiled at the port of landing, or at the port of embarkation; or we can go a step or two farther back and insist that the original purchaser and collector of rags shall be compelled to boil his little stocks and to deliver them to the rag merchant in a sweet and wholesome condition. One thing is quite certain, and that is that the rags have to pass through thoroughly purificative processes before they are remanufactured into paper and other goods. Why then should not the first stage of purification be carried out before they leave the hands of the original collector who gathers them in country villages and in the areas of the streets of large towns? Of course, there are practical difficulties in the way of carrying out such suggestions as these. But the question we have to ask is, not are there difficulties, but are there impossibilities? There is nothing like impossibility in the whole subject. The real point is this, would it be an advantage of the very first order of sanitary importance if wholesome rags alone were allowed for shipment, and if pestiferous rags were forbidden to be carried by sea, or, at any rate, landed at any British port? The answer admits of no doubt. The whole question is part of a larger question. But the larger question must also be dealt with sooner or later, and the sooner the better. Lord Beaconsfield was a much more prescient prophet than he himself knew when he said, "*Sanitas Sanitatum omnia Sanitas.*"

